# A New Double-Inclination Oblique Model to Simulate Drilling of GFRP/Al-Based Stacks: A Thermomechanical Approach

**DOI:** 10.3390/polym17081047

**Published:** 2025-04-12

**Authors:** Brahim Salem, Ali Mkaddem, Malek Habak, Yousef Dobah, Abdessalem Jarraya

**Affiliations:** 1 Laboratoire de Mécanique, Modélisation et Productique (LA2MP), National School of Engineering of Sfax, University of Sfax, Sfax 3038, Tunisia; salemiset@gmail.com; 2 Department of Mechanical and Materials Engineering, FOE, University of Jeddah, Jeddah 21589, Saudi Arabia; ydobah@uj.edu.sa (Y.D.); ajarraya@uj.edu.sa (A.J.); 3 Laboratoire Roberval, Centre de Recherche de Royallieu, Université de Technologie de Compiègne, Rue du Docteur Schweitzer, CS 60319, 60203 Compiègne, France; malek.habak@utc.fr

**Keywords:** GFRP/Al, oblique cutting configuration, temperature, drilling, VUMAT, VDFLUX

## Abstract

This paper reports an investigation of the thermomechanical behavior at the interface of GFRP/Al composite stacks when the stacking arrangement varies. A temperature-coupled damage approach was developed to simulate thermal energy transfer and damage propagation at metallic-to-composite interface. The proposed model was then implemented into ABAQUS/Explicit finite element code using user-defined subroutine VUMAT finely imbricated with VDFLUX. Unlike to previous models, oblique cutting configuration (OCC) involving double-inclination of the tool was proposed to simulate finely the material removal process owing to drill action. Drilling trials involving the cutting speed and the stacking arrangement were conducted to support the proposed approach. The predictions revealed that increasing the spindle speed significantly impacts the temperature distribution and subsurface thermal damage. An exponential temperature law was derived for predicting temperature variation with the cutting speed and identifying thermal saturation at the interface. The sensitivity of the composite behavior to the stacking arrangement (GFRP → Al vs. Al → GFRP) was well highlighted. The results indicated that attacking the structure from the GFRP side results in higher interfacial temperatures due to GFRP’s lower thermal conductivity. These findings contribute to understanding the heat-affected zone in GFRP, and, hence, provide guidance to minimize thermal damage in industrial drilling of the hybrid stacks.

## 1. Introduction

Nowadays, reducing structural weight is a key approach to enhancing aircraft performance and achieving minimum energy consumption. Consequently, demand for lightweight materials with high-performance properties has risen significantly in the aerospace industry over recent decades [1]. Glass fiber-reinforced polymer associated with aluminum alloy (GFRP/Al) structures become increasingly used in the emerging sectors attributable to their complementary properties [2]. However, most of the hybrid stacks are temperature-sensitive when subjected to loadings which leads to irreversible damage specially in the composite phase and at the stack interface, due to heat generation [3]. For assembling purposes, drilling is typically applied to prepare holes in composite structures for several engineering applications. Drilling of composite stacks involves different mechanisms compared to isotropic and homogeneous materials [4]. Such operation can lead to a range of defects such as thermal damage causing material degradation, delamination, fiber breakage, fiber pullout, matrix cracking, and debonding [5]. Consequently, controlling the cutting temperature together with the chip formation process is a critical challenge when drilling GFRP/Al components. If the cutting temperature exceeds the glass transition temperature $\left(T_{g}\right)$, permanent transformations luckily take place and material properties will be substantially lost [6], which affects directly the resistance of parts.

Single-shot drilling is typically suggested for manufacturing GFRP/Al stacks to reduce hole coaxiality errors and machining costs [7]. However, the significant difference in mechanical and physical properties of the constitutive materials makes the single-shot drilling a serious challenging task [8]. In such operation, the stacking arrangement is depicted among the critical parameter dominating the thermomechanical behavior of the constitutive phases.

In drilling hybrid GFRP/Al stacks, two common cutting strategies are generally implemented based on the tool engagement and stacking arrangement, i.e., GFRP → Al or Al → GFRP. Undoubtfully, the cutting strategies influence the integrity of the structure at exit and entry of the hole. While Al → GFRP strategy favors the extent of burr formation on the Al phase, GFRP → Al strategy seems to effectively reduce the exit burr height on the Al phase [9]. Beuscart et al. [10] studied the effect of the drilling sequence on the surface integrity when drilling GFRP/Al stacks using solid carbide drills with a six-axis robot. The study emphasizes how cutting conditions, especially the feed rate and cutting speed, affects drilling forces and hole quality within the stack in correlation with those obtained within the constitutive phases drilled separately. The authors tried to propose optimal process parameters for engineering applications. When studying carbon fiber-reinforced polymer and titanium stack (CFRP/Ti), Li et al. [11] reported that changing the cutting sequence from CFRP/Ti to Ti/CFRP enhances damage at the interface by effect of the metallic chip. The former can scratch the CFRP fresh surface, cause subsurface damage, and produce larger burrs at the interface.

It is worth noting that cutting conditions such as the cutting speed, feed rate, fiber orientation, tool geometry, stacking arrangement, and lubrication might also unfavorably alter the heat generation within the stack structures. Recent studies on drilling GFRP/Al have focused on minimizing heat generation, optimizing drilling parameters, and analyzing surface integrity [12,13,14,15]. As obvious, the drilling temperature was highlighted as critical factor that influences the hole quality in such structures. During the drilling step, it was proved that the temperature evolution and distribution depend sensitively on the thermal properties i.e., thermal conductivity and heat capacity, of the constitutive phases [16].

Wei et al. [17] investigated the machinability of CFRP/Ti basing on real-time temperature recordings using thermocouples pre-installed in the drill device. Within the CFRP phase, it was observed that the drilling temperature drops as the feed rate increases, while it remains unaffected by the variation of the cutting speed. Conversely, the temperature of the Ti phase increases in correlation with the increase in both the feed rate and cutting speed. As obvious, single-shot drilling of composite stacks yields complex heat transfer due to the discontinuity in thermal conduction at the interface between the constitutive layers [18]. Ge et al. [19] examined the cutting temperatures of CFRP, Ti alloy, and the stack interface in dry cutting, minimum quantity lubrication (MQL), and cryogenic cooling. It was revealed that the MQL environment enhances the efficiency of the cutting tool. Sun et al. [20] demonstrated that the interface temperature owing to helical milling significantly decreases compared to traditional drilling of Ti/CFRP/Al stack structure.

Experimental analyses basing on temperature measurement still remain costly, consuming time to build and to calibrate, and mostly limited because of local recordings. However, finite element analysis (FEA) constitutes a cost-efficient alternative, capable of modeling the material removal process in correlation with thermal behavior evolution of GFRP/Al composites. Several studies have proposed to simulate cutting of aluminum alloy or GFRP singly [13,21,22,23]. While the models predicting the cutting behavior of GFRP/Al stacks are limited, most of them omitted the thermal effects. Some studies have simplified the cutting process of GFRP/Al stacks to orthogonal or oblique cutting to examine the chip removal process and the mechanisms of damage formation [11,16]. In oblique cutting configuration, the angle of the chip flow is depicted as critical parameter. In such configuration, the orientations of both the cutting velocity and the chip flow are not orthogonal to the cutting edge [24]. Chang et al. [25] presented an energy-based mechanistic approach for predicting cutting forces in the drilling of SiCp/Al composites, incorporating an oblique cutting model. The model offers accurate cutting force predictions and estimates the energy consumed. As to Xu et al. [26], they presented a 3D finite element model to simulate oblique and sequential machining of CFRP, focusing on the effects of fiber orientation, oblique angle, and sequential cutting on chip formation, cutting forces, and surface damage. Increasing the oblique angle accentuates the out-of-plane forces and surface damage, while sequential cutting reduces forces in subsequent passes.

This attempt covers an advanced temperature-coupled damage approach to predict the effects of drilling-induced heat on individual phases within hybrid composites. Basing on oblique cutting configuration, the study examined the thermomechanical interactions within the GFRP/Al interface under different cutting conditions. Specified user-defined subroutines were implemented into ABAQUS/Explicit code to simulate heat generation and damage mechanisms. The new model offers critical insights into the influence of stacking arrangement on the thermal and mechanical responses at the interface of the hybrid composites. Ultimately, the approach is found very useful for deciding about machining strategies for such structures, with experimental validation confirming the model reliability.

## 2. Materials and Methods

### 2.1. Specimen Preparation and Drilling Conditions

Unidirectional glass/epoxy panels obtained by hand lay-up technique are considered together with Al2020 aluminum alloy for preparing the composite stack specimens. The glass/epoxy panels consisted of E-Glass cloth of $520\;g\cdot m^{-2}$ supplied by Castro Composites Co., Pontevedra, Spain, and 1050-grade epoxy resin supplied by Resoltech Co., Rousset, France. The resin integrated 35% (in volume) of 1055S-grade hardener. Thermomechanical analyses were conducted on both, GFRP/Al and Al/GFRP stacking arrangements.

**Figure 1:**
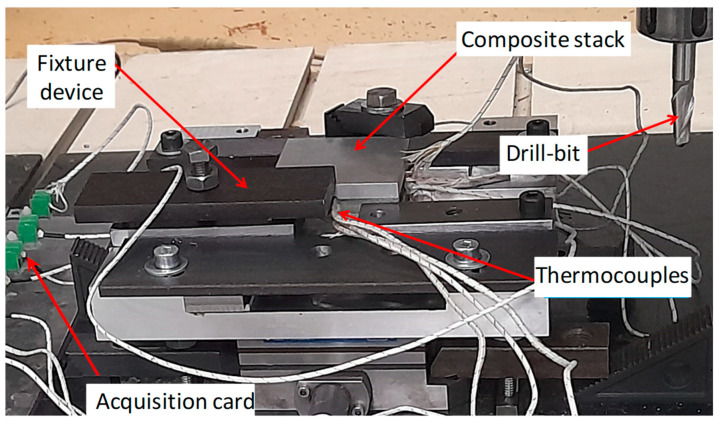
Experimental setup showing the specimen with the TCs placement.

The drilling tests were performed upon Charlyrobot CNC machines Model CPR0705 belonging to Mécanuméric, Marssac-Sur-Tarn, France. Figure 1 shows the experimental setup including the specimen and temperature acquisition device. K-type thermocouples (TCs) supplied by Electronic Shop Co., Sfax, Tunisia, capable of sensing from −270 °C to 1370 °C, were used to record temperature when drilling. The TCs sensing tips were preinstalled 1 mm away from the drilling wall, inside 2 mm-diameter holes. All cutting trials were conducted in dry conditions, at constant feed rate of 1 mm/s, using carbide drill of 6.3 mm diameter, 5° clearance angle, 150°- point angle, 30° helix angle, and 80 mm working length. The design of experiments included four cutting speeds, i.e., 71, 95, 119, and 142 m/min, and two stacking arrangement, namely, GFRP/Al and Al/GFRP.

### 2.2. Estimation of Heat Generation in Hybrid GFRP/Al

**Figure 2:**
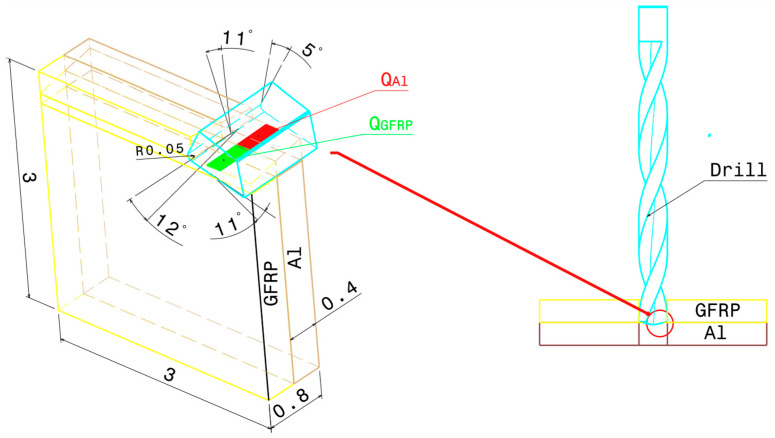
Thermomechanical model of oblique cutting in the drilling process.

In oblique cutting of hybrid GFRP/Al composites, a large portion of the energy is converted into heat. Therefore, the heat flow into the workpiece can be represented as follows:

(1) $$Q_{w}=Q_{GFRP}+Q_{Al}$$

where $Q_{GFRP}$ is the heat dissipated by the GFRP phase, and $Q_{Al}$ represents the heat dissipated by the aluminum phase. This distinction is essential, as the heat flux at the tool/GFRP interface differs from that at the tool/Al interface due to variations in friction across their respective contact areas (Figure 2).

**Figure 3:**
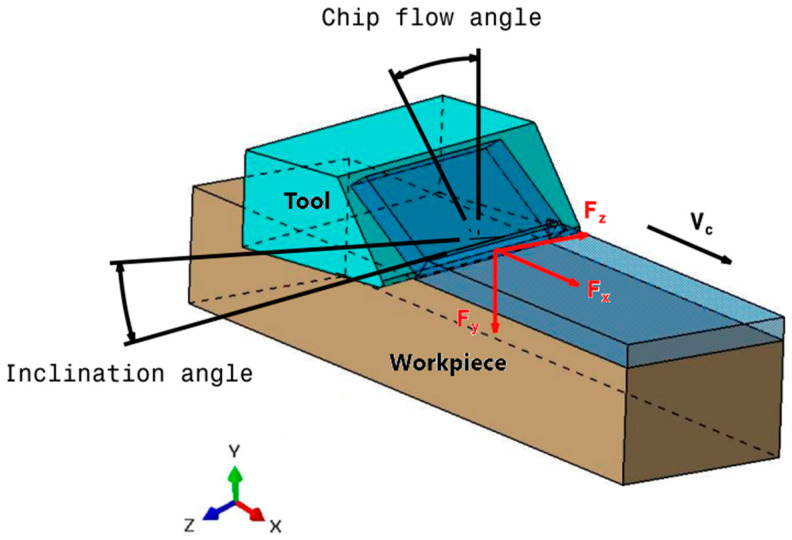
Schematic representation of inclination angle of the tool in oblique cutting.

Furthermore, the oblique nature of the cutting process results in three force components: $F_{x}$ (horizontal), $F_{y}$ (vertical), and $F_{z}$ (transverse) as depicted in Figure 3. Given that displacements caused by the vertical and transverse cutting forces are minimal, we focus solely on the work completed by the horizontal cutting force to determine the heat generation within the contact zone. This approach aligns with the methodologies employed in previous studies [18,27,28,29,30]. While the cutting force exhibited some variation throughout the cutting process, for the purpose of this analysis, we utilized the average value of the measured forces. This simplification allowed us to define the total heat flow generation within the primary shear zone during the machining process as follows:

(2) $$Q_{p}=F_{x}v_{c}\;$$

In the secondary shear zone, frictional interactions at the tool-material interface contribute to the overall heat generation. This heat flow can be defined as follows:

(3) $$Q_{s}=F_{f}v_{s}$$

where $Q_{s}$ is the frictional heat generated in the secondary shear zone, $F_{f}$ is the frictional force at the tool-material interface, and $v_{s}$ is the sliding velocity between the tool and the chip.

The total heat flow generated in the contact zone $Q_{total}$ can be represented as follows:

(4) $$Q_{total}=Q_{w}+Q_{tool}+Q_{chip}=F_{x}v_{c}+F_{f}v_{s}$$

where $Q_{w}$ is the heat flow transferred to the GFRP workpiece, $Q_{tool}$ is the portion of heat flow that flows into the cutting tool, and $Q_{chip}$ the heat flow carried away by the generated chip.

Here, we focus on the transfer of thermal flow into the workpiece, highlighting the effects of thermomechanical interactions on the structural integrity of the hybrid assembly. Therefore, only the $Q_{w}$ parameter is incorporated into ABAQUS/Explicit 3DEXPERIENCE R2017x, with the assumption that localization within the secondary shear zone has a negligible effect. This simplifies Equation (4) to

(5) $$Q_{w}+Q_{tool}=F_{x}v_{c}$$

### 2.3. Estimation of Heat Flux Applied to GFRP

In the oblique cutting of unidirectional GFRP composites, the anisotropic and inhomogeneous nature of the material plays a critical role in heat flux distribution within the workpiece. This anisotropy implies that the material properties vary with the fiber orientation, leading to a situation where the fiber angle θ significantly influences the amount of heat entering the workpiece. Specifically, the fiber orientation directly affects friction at the tool–workpiece interface, as different orientations cause varying levels of resistance and, therefore, differing frictional forces. This frictional variation is a key factor because it directly influences the cutting forces, which, in turn, are crucial components in calculating the heat flux. In oblique cutting of GFRP, the heat flux, $Q_{GFRP}$, applied to the workpiece is determined by

(6) $$Q_{GFRP}=\lambda_{GFRP}\frac{F_{xGFRP}\times v_{c}}{A_{GFRP}}$$

where $\lambda_{GFRP}$ represents the partition ratio of thermal flow that is transmitted into the workpiece [27], $F_{xGFRP}$ denotes the horizontal cutting force, and $A_{GFRP}$ indicates the contact area between the composite workpiece and the operational cutting tool as described by

(7)  AGFRP=90°+α0+γ0180°πRc+1sin α0·3Fy4E*Rc23·lGFRP

where $\alpha_{0}$ is the tool clearance angle, $\gamma_{0}$ is the rake angle of the tool. $R_{c}$ is the radius of the cutting edge of the tool, $F_{y}$ is the vertical cutting force, $l_{GFRP}$ is the thickness of the GFRP plate, and $E^{*}$ is the equivalent elastic modulus of the cutting tool is expressed as

(8) $$\frac{1}{E^{*}}=\left(\frac{1}{G_{12}}-2\frac{\upsilon_{21}}{E_{11}}\right)\cdot {sin}^{2}\theta \;{cos}^{2}\theta +\frac{\left(1-\upsilon_{12}^{2}\right){sin}^{4}\theta }{E_{11}}+\frac{{cos}^{4}\theta }{E_{22}}+\frac{1-\upsilon^{2}}{E}$$

where θ denotes the orientation of the fiber, $G_{12}$ represents the shear modulus of the composite material, $E_{ii}$ signifies the elastic modulus of the composite material in the i-direction, $\upsilon_{ij}$ refers to the Poisson’s ratio of the composite material concerning transverse strain in the j-direction when the applied stress in i-direction, and υ and E correspond to the Poisson’s ratio and the elastic modulus of the tool, respectively.

### 2.4. Estimation of Heat Flux Applied to Aluminum

In the oblique cutting process involving metals, thermal flow is disseminated across the primary, secondary, and tertiary shear zones. According to an extensive literature review, the ratios of heat partitioning directed towards the cutting tool, the workpiece, and the chip were observed to vary between 0.02 and 0.18, 0.01 and 0.2, and 0.74 and 0.96, respectively [31,32]. In oblique cutting of aluminum, the heat flux, $Q_{Al}$, applied to the workpiece is determined by

(9) $$Q_{Al}=\lambda_{Al}\frac{F_{xAl}\times v_{c}}{A_{Al}}$$

where $\lambda_{Al}$ is the partition ratio of the thermal flow that is introduced into the workpiece (0.2) [31,32], $F_{xAl}$ represents the horizontal cutting force, $v_{c}$ signifies the cutting velocity, and $A_{Al}$ is the effective area of aluminum phase at the primary shear zone.

## 3. Constitutive Approach

### 3.1. Heat Transfer Model for Hybrid Composite

The transient thermal transfer phenomenon occurring during the orthogonal machining of hybrid GFRP/Al composites is articulated in accordance with the heat conduction equation, expressed as follows:

(10) $$\lambda_{11}\frac{{𝜕}^{2}T}{𝜕x^{2}}+\lambda_{22}\frac{{𝜕}^{2}T}{𝜕y^{2}}+\lambda_{33}\frac{{𝜕}^{2}T}{𝜕z^{2}}+q\left(x,y,z\right)=\rho c\frac{𝜕T}{𝜕t}$$

where $\lambda_{11}$ denotes the thermal conductivity aligned with the fiber orientation, while $\lambda_{22}$ and $\lambda_{33}$ represent the thermal conductivities in the orthogonal directions, q signifies the heat flux, and T indicates the incremental rise in temperature.

### 3.2. GFRP Constitutive Behavior

#### 3.2.1. Temperature-Dependent Properties

The thermo-physical properties of unidirectional GFRP composites are notably sensitive to temperature increases caused by the cutting actions. This sensitivity necessitates adjustments in material property models during machining to ensure accuracy. Therefore, the model developed by Gibson et al. [33], which utilizes a hyperbolic tangent function, has been adopted as a reliable approach for keeping composite properties current throughout the machining process. This model, designed to account for real-time variations in thermal behavior, provides an effective way to capture the dynamic responses of GFRP composites as they undergo thermal changes. The model’s formulation is presented as follows:

(11) $$P\left(T\right)=\frac{1}{2}\left(P_{0}-P_{r}\right)tanh\left[-\frac{1}{\Delta T/2}\left(T-\left(T_{g}+\frac{\Delta T}{2}\right)\right)\right]+\frac{1}{2}\left(P_{0}+P_{r}\right)$$

In this context, P represents the mechanical characteristic at a specific temperature T, $P_{0}$ signifies the mechanical characteristic at ambient temperature, and $P_{r}$ indicates the relaxed (elevated temperature) value of the mechanical characteristics. $T_{g}$ denotes the glass transition temperature, while ΔT refers to the temperature deviation from the glass transition point of the polymeric matrix.

#### 3.2.2. Damage Initiation Criteria

In composite materials, particularly unidirectional fiber-reinforced composites, damage initiation signifies the point at which material degradation begins due to stress or strain accumulation. Various failure criteria have been developed to predict this initiation point, including the stress or strain criteria, Tsai-Hill, and Tsai-Wu criteria. Among these, Hashin’s criteria is widely recognized due to its specificity in identifying the relevant damage mode for each phase within the laminate and corresponding to each loading scenario. This distinction allows Hashin’s criteria [34] to account for discriminating failure mechanisms in the fiber and matrix phases, making it more sensitive to the distribution and magnitude of stress within the composite. Once a criterion threshold is met, the material properties at that point degrade according to the specific failure mode. This degradation in material properties, reflecting the onset of damage, leads to an update in the local stress distribution, simulating the composite’s weakened structural response. Up until the onset of damage, the composite’s behavior remains linear elastic, ensuring accurate predictions for the point of failure. The corresponding failure criteria for these damage modes are summarized in the following Equations (12)–(15).

For fiber tension ($\sigma_{11}\geq 0$),

(12) $${\left(\frac{\sigma_{11}}{X_{T}}\right)}^{2}+{\left(\frac{\sigma_{12}}{S_{12}}\right)}^{2}+{\left(\frac{\sigma_{13}}{S_{13}}\right)}^{2}=\left\{\begin{array}{c} F_{f}^{t}\geq 1\;failure\;\;\\ F_{f}^{t}<1\;nofailure \end{array}\right.$$

For fiber compression ($\sigma_{11}\leq 0$),

(13) $${\left(\frac{\sigma_{11}}{X_{C}}\right)}^{2}=\left\{\begin{array}{c} F_{f}^{c}\geq 1\;failure\;\;\\ F_{f}^{c}<1\;nofailure \end{array}\right.$$

For matrix tension ($\sigma_{22}+\sigma_{33}\geq 0$),

(14) $$\frac{{\left(\sigma_{22}+\sigma_{33}\right)}^{2}}{{Y_{T}}^{2}}+\frac{{\sigma_{23}}^{2}-\sigma_{22}\sigma_{33}}{{S_{23}}^{2}}+\frac{{\sigma_{12}}^{2}+{\sigma_{13}}^{2}}{{S_{12}}^{2}}=\left\{\begin{array}{c} F_{m}^{t}\geq 1\;failure\;\;\\ F_{m}^{t}<1\;nofailure \end{array}\right.$$

For matrix compression ($\sigma_{22}+\sigma_{33}\leq 0$),

(15) $$\left[{\left(\frac{Y_{C}}{2S_{23}}\right)}^{2}-1\right]\frac{\left(\sigma_{22}+\sigma_{33}\right)}{Y_{C}}+\frac{{\left(\sigma_{22}+\sigma_{33}\right)}^{2}}{4S_{23}^{2}}+\frac{{\tau_{23}}^{2}-\sigma_{22}\sigma_{33}}{S_{23}^{2}}+\frac{{\sigma_{12}}^{2}+{\sigma_{13}}^{2}}{S_{12}^{2}}=\left\{\begin{array}{c} F_{m}^{c}\geq 1\;failure\;\;\\ F_{m}^{c}<1\;nofailure \end{array}\right.$$

In the preceding equations, $\sigma_{ij}\left(i,j=1,2,3\right)$ represents the effective stress tensor components, $X_{T}$ and $Y_{T}$ are the tensile failure stresses in longitudinal and transverse direction, and $X_{C}$ and $Y_{C}$ are the compressive failure stresses in longitudinal and transverse direction, respectively. $S_{ij}\left(i,j=1,2,3\right)$ are components of the shear failure stresses in-plane and out-of-plane.

#### 3.2.3. Damage Evolution

In the damage evolution model, the progression of degradation in both fibers and the matrix are governed by an exponential law based on the function proposed by Matzenmiller et al. [35]. This formulation captures the damage variables for fiber and matrix through a function that expresses them as

(16) $$d_{i}^{j}=1-exp\left(\frac{1}{m_{i}^{j}}{\left(1-F_{i}^{j}\right)}^{m_{i}^{j}}\right)$$

where $m_{i=f,m}^{j=t,c}$ are the material softening parameters that determine the rate of damage for each specific loading condition. In line with the findings of Khan et al. [36], these parameters, $m_{f}^{t},\;m_{f}^{c},m_{m}^{t},m_{m}^{c}$, are calibrated to values of 0.2, 0.07, 1.15, and 3, respectively. Once the damage parameters are obtained, the stresses can be updated as

(17) $$\left[\sigma^{n}\right]=\left[C^{n}(d^{n-1})\right]\left[\varepsilon^{n}\right]$$

where $\sigma^{n}$ is the stress tensor and $\varepsilon^{n}$ the strain tensor whose components are updated according to the strain increment Δε calculated at each iteration as,

(18) $$\varepsilon^{n}=\varepsilon^{n-1}+\Delta \varepsilon^{n}$$

**Figure 4:**
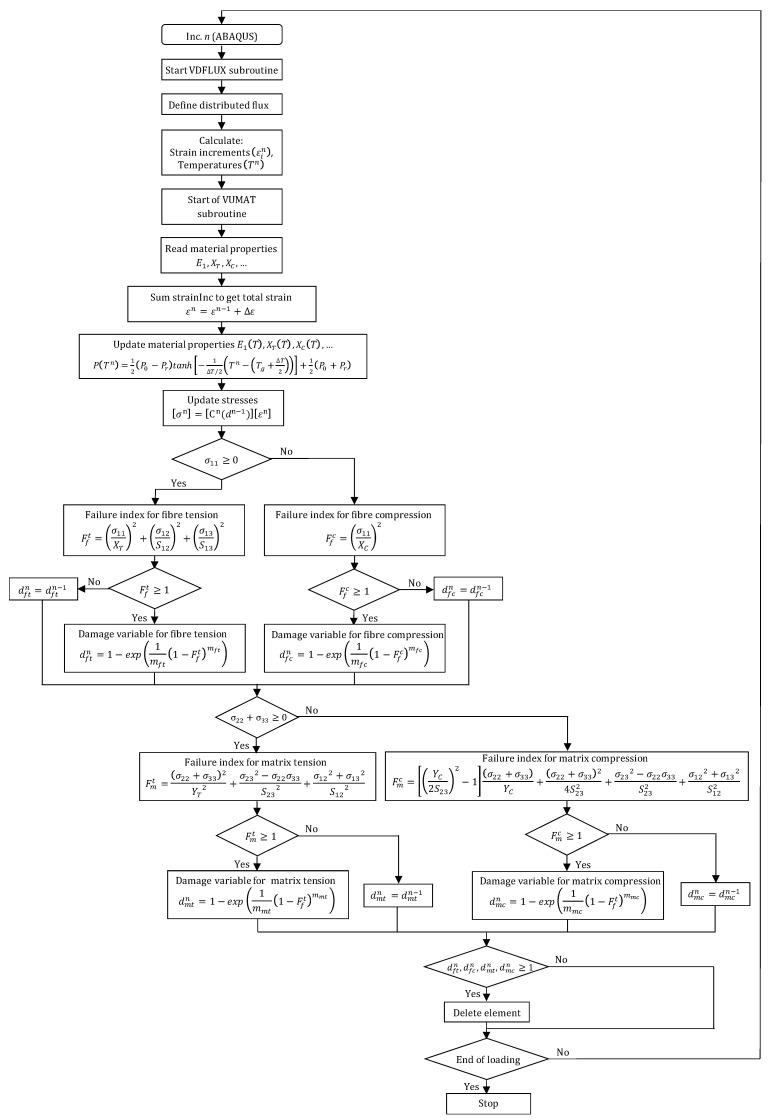
Numerical algorithm describing temperature-coupled damage approach for composite behavior.

Figure 4 illustrates the incremental scheme of temperature-coupled damage algorithm. The proposed FE model concerns a temperature-coupled damage approach capable of updating temperature within each phase of the composite structure i.e., GFRP, Al, and the interface, at each increment. The development associates finely two user-defined subroutines. The VUMAT which manages the stress-to-strain relationship with regard to damage initiation described by Hashin’s criteria, and damage evolution described by Matzenmiller’s law. The VDFLUX which manages temperature evolution and localization sensitively to the heat flux applied at the fresh surface of the composite stack.

### 3.3. Aluminum Constitutive Behavior

In relation to the aluminum phase, the Johnson–Cook constitutive model and damage criteria [37,38] were employed to characterize the material’s response under high plastic deformation and elevated strain rates during the machining process. The equivalent flow stress was determined utilizing material constants as follows:

(19) $$\bar{\sigma }=\left[A+B{\left(\bar{\varepsilon }\right)}^{n}\right]\left[1+Cln\left(\frac{\dot{\bar{\varepsilon }}}{{\dot{\bar{\varepsilon }}}_{0}}\right)\right]\left[1-{\left(\frac{T-T_{0}}{T_{m}-T_{0}}\right)}^{m}\right]$$

where $\bar{\sigma }$ represents the equivalent flow stress, $\bar{\varepsilon }$ denotes the equivalent plastic strain, $\dot{\bar{\varepsilon }}$ signifies the equivalent plastic strain rate, ${\dot{\bar{\varepsilon }}}_{0}$ refers to the reference equivalent plastic strain rate, T indicates the effective temperature attained in the titanium phase, $T_{m}$ corresponds to the material’s melting temperature, and $T_{0}$ represents ambient room temperature. *A*, *B*, *C*, *m*, and *n* are material constants that necessitate empirical determination. The Johnson–Cook damage criteria are also integrated into the aluminum phase to effectively model the material removal process. The equivalent plastic strain at the onset of damage [36,37] is expressed in Equation (1),

(20) $$\bar{\varepsilon_{0}}=\left[d_{1}+d_{2}exp\left(d_{3}\frac{P}{\bar{\sigma }}\right)\right]\left(1+d_{4}ln\frac{\dot{\bar{\varepsilon }}}{\dot{\bar{\varepsilon_{0}}}}\right)\left[1+d_{5}\left(\frac{T-T_{r}}{T_{m}-T_{r}}\right)\right]$$

where $\varepsilon_{0}$ denotes the plastic strain at the onset of damage, P signifies the hydrostatic pressure, and $d_{i}$, where i = 1 … 5, represents the failure constants to be determined experimentally using notched and axisymmetric specimens under various stress states, temperatures, and strain rates. Thus, the separation of the chip transpires when $\bar{\varepsilon_{0}}$ attains the plastic strain threshold, and the fracture criteria delineated below, reaches one (*f = 1*):

(21) $$f=\frac{\sum \bar{\varepsilon }}{{\bar{\varepsilon }}_{0}}$$

All numerical parameters were meticulously delineated to characterize the Johnson–Cook constitutive model as well as the damage model. Initial simulations were performed to replicate the cutting process of the aluminum phase singly, and the efficacy of the user-defined material in forecasting the mechanisms of chip formation was convincingly validated.

### 3.4. Oblique Cutting Geometry

The drilling operation can be realistically modeled as oblique cutting configuration due to the inclination angle of the cutting tool and the axial feed advance (Figure 3). This inclination creates a complex interaction between the tool and the workpiece, influencing the direction of chip flow and the forces involved. The combined effect of the tool’s tilt and the feed direction leads to a three-dimensional cutting action. The current approach aims to determine the inclination angles used in the oblique cutting model based on the drill geometry considered in the drilling tests.

According to Chang [25], certain parameters on the major cutting edges such as cutting speed $\left(v_{c}\right)$ and inclination angle $\left(\lambda_{s}\right)$, change when drilling as the radial distance varies. The inclination angle $\lambda_{s}$ is expressed as function of tool geometry as

**Figure 5:**
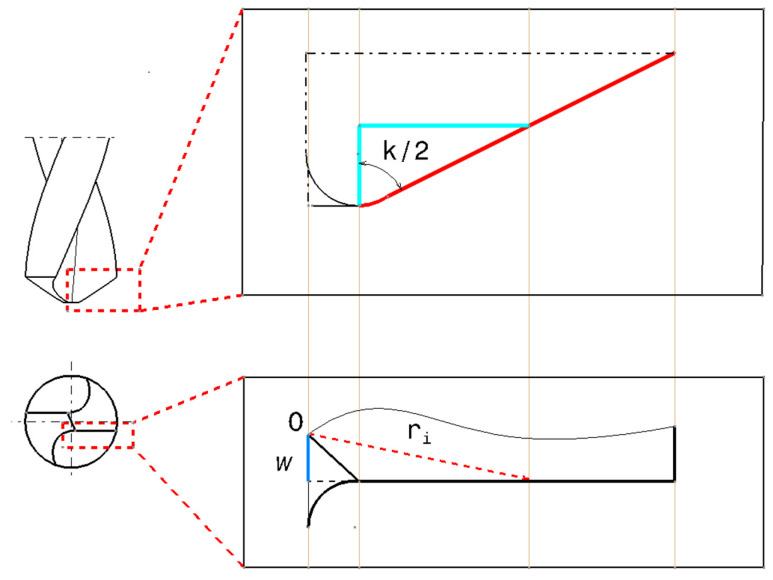
Geometrical relationship of radial distance along the cutting edge.

(22) λs=arcsinωri sink2

where ω is the half of the thickness of the chisel edge, $r_{i}$ is the radial distance of the drill, and k is the drill point angle. Figure 5 illustrates the geometrical characteristics of the drill.

As for the chip flow angle, $\mu_{c}$, it can be obtained as specifically described by Stabler [39], based on the following relation:

(23) $$\mu_{c}=0.9\lambda_{s}$$

### 3.5. Finite Element Model

A three-dimensional damage model was incorporated into the finite element analysis software via a user-defined subroutine, VUMAT, which is accessible within the ABAQUS/Explicit code. This enabled the prediction of the extent and mode of damage within each phase of the hybrid structure, based on the type of loading applied to the targeted finite element. The damage modeling accounts for a complete three-dimensional stress state. The temperature-coupled displacement methodology was executed through a user-defined VDFLUX, which serves to meticulously adjust the material properties in response to temperature variations. A moving surface heat flux was applied at both the trim plane and the tool-material interface to simulate the temperature generated by the cutting process. The credibility of the proposed model in simulating the machining of hybrid materials, specifically GFRP/Al, was evaluated through a comparison of both the predicted and experimentally measured. The cutting parameters, along with the characteristics of the tool and workpiece (including depth of cut, cutting speed, rake angle, clearance angle, and edge radius), were systematically delineated in conjunction with the experimental work for validation purposes.

It is important to highlight that the cumulative heat produced during the machining process was determined based on the available experimental data, and the heat ratio to be transferred into the workpiece served as an essential input for the model. The objective of the model is to forecast the implications of temperature distribution on the structural integrity of GFRP/Al composites. The characteristics of the GFRP phase necessary for the user-defined subroutines are delineated in Table 1.

The parameters employed according to the Johnson–Cook constitutive framework (Equation (1)) and the damage model (Equation (2)) are documented in Table 2, along with the mechanical attributes of the aluminum alloy.

The specifications of the tool, the geometry of the workpiece, and the parameters associated with cutting are delineated in Table 3. The workpiece was conceptualized as a prismatic component, comprising both aluminum and GFRP phases of identical width $\left(w_{GFRP}=w_{Al}=0.4\mathrm{mm}\right)$. The tool, characterized as a completely rigid dody, is permitted movement solely in the direction of cutting, while all degrees of freedom are entirely constrained at the inferior surface of the workpiece. The contact interaction occurring at the interface between the tool and the workpiece was simulated utilizing the surface–node surface contact algorithm provided in ABAQUS/Explicit, employing a fixed friction coefficient value set at 0.3.

**Figure 6:**
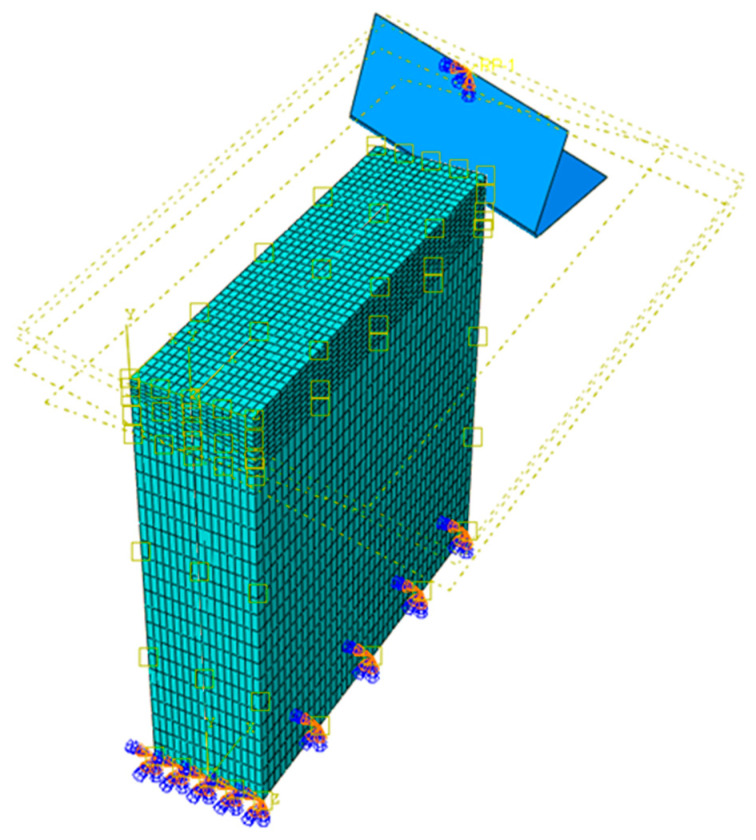
FE model proposed for the oblique cutting configuration.

The proposed model excludes consideration of the interfacial layer, as the constituents of the stack are only in contact during the actual processing phase. The “Tie” property was employed to articulate the interaction conditions between the phases of the stack. The mesh of the workpiece was meticulously refined within the cutting zone using a finite element size of 40 µm. Eight-node thermally-coupled displacement brick element (C3D8T) was used to effectively capture temperature variations throughout the cutting operation. Figure 6 provides the details of the geometry, initial tool placement, and boundary conditions adopted in the oblique cutting model.

## 4. Results and Discussion

### 4.1. Impact of Stacking Arrangement and Cutting Speed on GFRP/Al Interface Temperature

In hybrid GFRP/Al drilling, two main cutting strategies are employed, i.e., GFRP → Al and Al → GFRP, depending on the tool’s engagement in the structure. This choice of cutting arrangement greatly influences the final drilling temperatures in the bi-material system.

#### 4.1.1. GFRP → Al Cutting Mode

**Figure 7:**
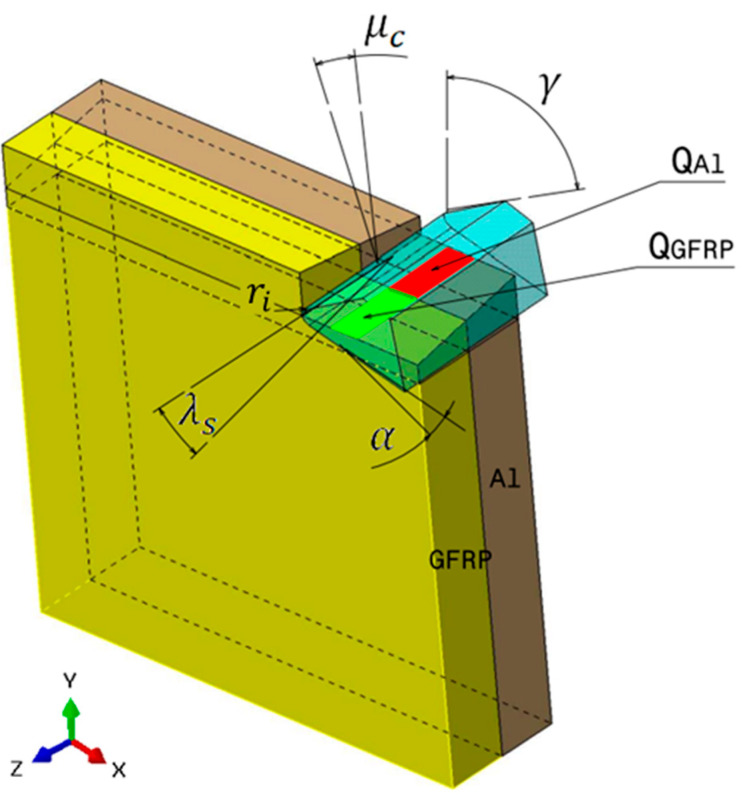
Oblique cutting—Side attack configuration for the GFRP phase.

In GFRP → Al configuration, the rotation of the tool $\mu_{c}$ is along the +x axis (positive inclination angle), while the rotation $\lambda_{s}$ is along the +y axis (positive inclination angle). Figure 7 illustrates the oblique cutting model, depicting an approach on the hybrid material from the GFRP composite phase side.

In the oblique cutting process of a GFRP/Al stack, the thermomechanical interaction between the aluminum and GFRP phases significantly affects the temperature at the interface. As shown in Table 4, with identical tool specifications and cutting parameters, the temperature in the aluminum and GFRP phases is notably influenced by variations in the cutting speed. The cutting temperature of the GFRP plate consistently reaches its maximum at the interface, independent of the cutting speed. Nevertheless, the aluminum phase exhibits a nearly homogenous thermal distribution throughout its entire thickness. The simulation proficiently emulates the chip formation mechanisms associated with the hybrid constituents, precisely forecasting the occurrence of continuous chips within the aluminum phase and the presence of fragmented chips in the glass fiber-reinforced polymer (GFRP) phase. The chip produced from the GFRP phase is not visible because the “element deletion” option was applied to simulate material removal once any damage criteria was met. 

An analysis of the predicted fields on the GFRP interface side shows that, even after the tool passes, the temperature remains concentrated at relatively high levels along the edge shared by the trim plane and the interface.

The isovalues were recorded with reference to the glass transition temperature of the GFRP $\left(T_{g}=70\;{}^{\circ}C=343K\right)$. The results revealed the thermally affected zone, particularly at the interface between the two phases. In the Al phase, the predictions showed an increase in the area affected on the fresh surface with the cutting speed (gray zone). This effect extends to the GFRP plate interface, where the critically affected depth (area where $\left(T>T_{g}\right)$ increases with the cutting speed.

#### 4.1.2. Al → GFRP Cutting Mode

**Figure 8:**
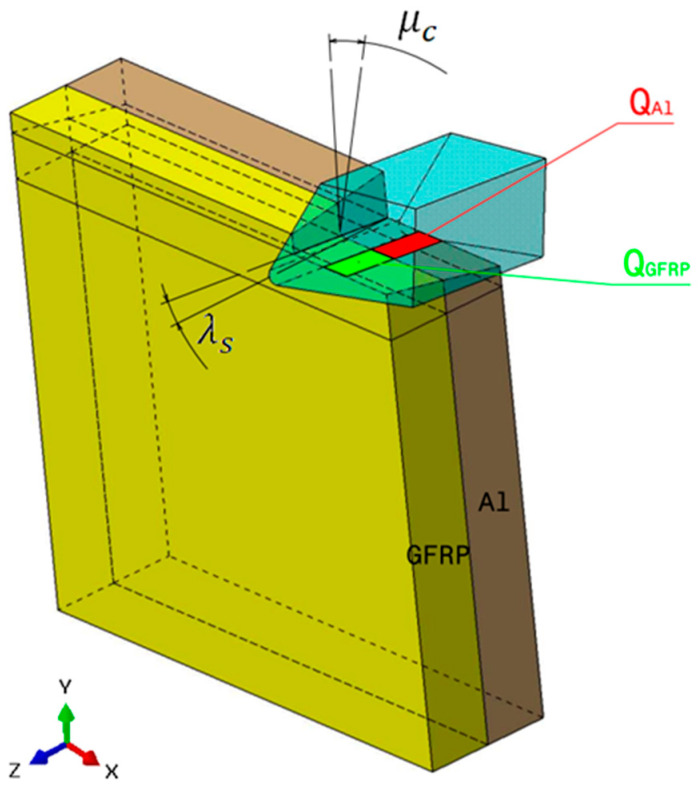
Oblique cutting—Side attack configuration for the Al phase.

In Al → GFRP configuration, the rotation of the tool $\mu_{c}$ is along the -x axis (negative inclination angle), while the rotation $\lambda_{s}$ is along the -y axis (negative inclination angle). Figure 8 illustrates the oblique cutting model, depicting an approach on the hybrid material from the GFRP composite phase side.

As shown in Table 5, when cutting from the aluminum layer, the temperature increases with the cutting speed, reaching its peak at the maximum speed used in both constituent phases. Consequently, at the interface, the critically affected depth appears to increase significantly with the cutting speed.

When confronting the predictions of the two cutting cases (Table 4 and Table 5), the maximum temperatures seem to be similar, except in the case of the cutting speed $v_{c}=142\;m\cdot \min^{-1}$. The maximum cutting temperature is higher when cutting from the GFRP layer than when drilling from the aluminum layer. This is mainly attribute to the thermal conductivity of GFRP which is significantly lower to that of aluminum. Beyond the threshold temperature $T_{g}=343K$ the matrix phase of the GFRP composite may undergo localized burning, as indicated by the gray contours in both GFRP → Al and Al → GFRP cutting cases. However, in the first case, the heat-affected zone extends along the cutting plane in a uniform manner with increasing cutting speed, remaining limited to the interface, even as the temperature in the Al phase exceeds $T_{g}$ over almost the entire fresh surface.

It is clear that the heat generated by the flow of metal chips dominates the thermal process at the interface, where the critical temperature is transferred to the GFRP phase. Thus, the interface is subject to competing thermal mechanisms: a dominant heat flow from the Al phase working to transmit heat toward the GFRP phase, and a less dominant heat flow from the GFRP phase working to limit the critically affected area and slow heat propagation into the composite phase. This, of course, is closely related to the thermal properties of each phase, particularly thermal conductivity. In the Al → GFRP cutting case, a similar heat localization is observed, though the affected area is less uniform and appears to evolve less significantly with the cutting speed compared to the previous case.

### 4.2. Effect of Cutting Speed on Temperature Evolution

**Figure 9:**
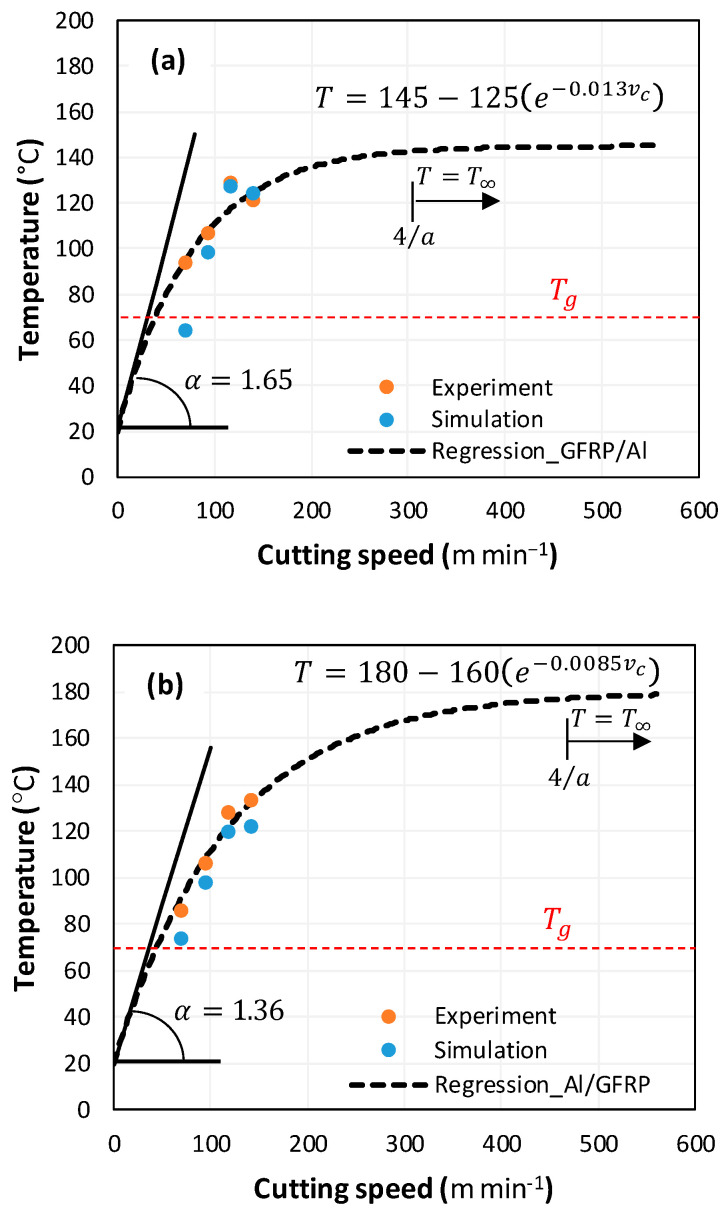
Interface temperature vs. cutting speed. (**a**) GFRP/Al stack, and (**b**) Al/GFRP stack.

To study the validity of the predictions obtained through the approach used in this work, temperature measurements were carried out through instrumented tests using thermocouples (TCs) pre-installed at the interface and within the phases of the GFRP/Al and Al/GFRP stacks. The thermocouples (TCs) were placed at different positions around the hole to be drilled. At all measurement points, the temperature was recorded 1 mm away from the finished surface. The measurement results were then extrapolated to the surface for comparison with the predictions, which were recorded directly at the fresh surface. Figure 9 presents the numerical and experimental results associated with drilling the hybrid composite.

The curves reflect the effect of the cutting speed on the temperature evolution. Overall, the temperature evolution was similar in both cases, with good agreement between the experimental results and the numerical predictions. The evolution was clearly nonlinear and is represented by an exponential law of the form:

(24) $$T=A-Be^{-a.v_{c}}$$

where A is the temperature at infinity $\left(A=T_{v_{c\to \infty }}=T_{\infty }\right)$, B is the temperature variation margin relative to the reference temperature $T_{0}$, such that $B=T_{\infty }-T_{0}$, and a is a constant to be determined experimentally.

The maximum values were recorded for the highest cutting speed. Comparing the two figures, the discrepancy between the predictions fluctuated between 6.7 and 16.5%. However, the simulation appeared to underestimate the temperature, showing standard deviations between the predictions and the experimental results of approximately 8.5 ± 3.17 °C for the GFRP → Al cutting case, and around 6.0 ± 1.72 °C for the Al → GFRP cutting case.

Smoothing and extrapolation of the experimental results using exponential regression beyond the maximum cutting speed predicted a convergence of the temperature to a stable value as the speed increases. The temperature stabilized at $T_{\infty }\pm 2\%$ starting at a cutting speed of approximately $v_{c}=\frac{4}{a}$, regardless of the arrangement of the constituent phases. The temperature stabilized at a speed of around 308 m/min when cutting through the composite. However, in the case of cutting through aluminum, the stabilization plateau was reached at a speed of around 471 m/min. This demonstrates the influence of the temperature evolution rate, which allowed for faster thermal saturation (145 °C) in the case of cutting through the GFRP phase, while the maximum temperature value was lower than in the case of cutting through the Al phase (180 °C).

The slope at the origin of the exponential law provides information on the rate of temperature change at the interface at the start of the drilling process. It can be obtained by

(25) $${\left.\alpha =\frac{dT}{dv_{c}}\right|}_{v_{c}=0}={\left.B.a.e^{-a.v_{c}}\right|}_{v_{c}=0}=B.a$$

According to the calculations for the value of α, the temperature rises more rapidly with the cutting speed in the case of the hybrid GFRP/Al ($\alpha_{GFRP/Al}=1.65>1.36=\alpha_{Al/GFRP}$). This leads to thermal saturation being reached at a threshold cutting speed $\left(v_{c}^{s}=\frac{4}{a}\right)$ that is relatively lower than in the case of the Al/GFRP $\left(v_{c-Al/GFRP}^{s}=1.5\times v_{c-GFRP/Al}^{s}\right)$, where $v_{c}^{s}$ is the threshold cutting speed indicating the onset of thermal saturation. The proposed exponential law is an excellent indicator of thermal behavior as it enables the prediction not only of the saturation temperature but also of the thermal kinetics at the composite–metal interface.

### 4.3. Damage Contours Analysis

**Figure 10:**
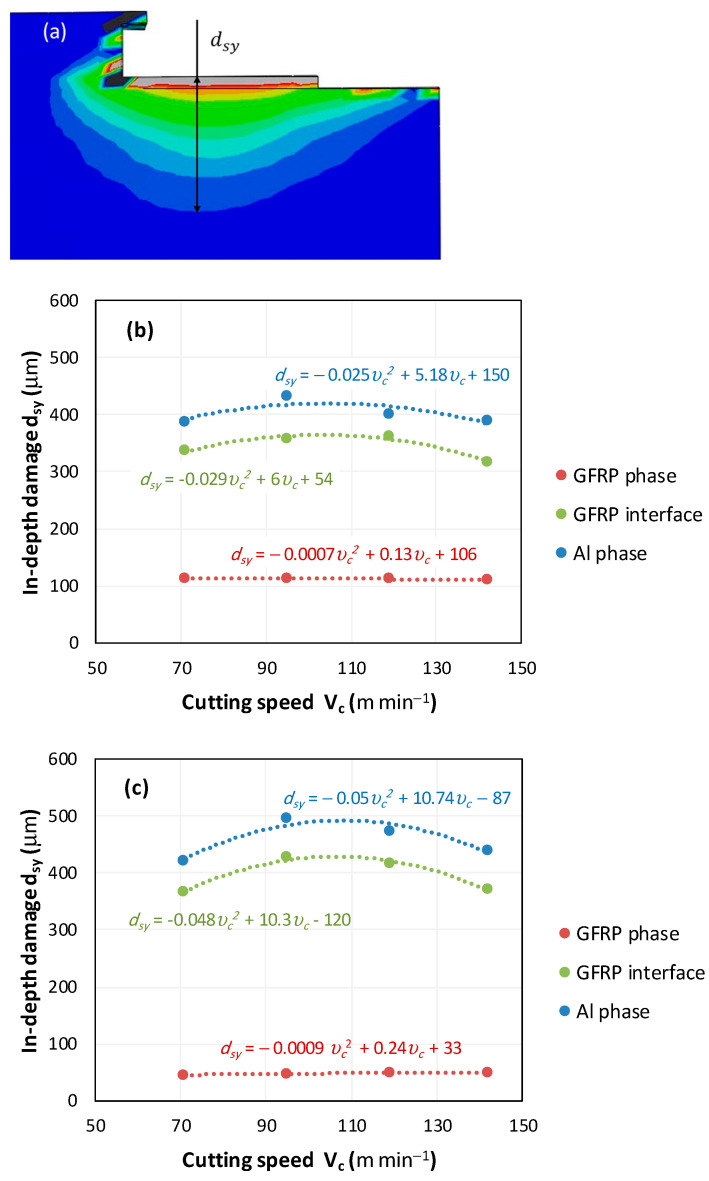
Subsurface damage evolution vs. cutting speed. (**a**) Damage measure description, (**b**) in-depth damage GFRP/Al stack, and (**c**) in-depth damage Al/GFRP stack.

It would be interesting to examine subsurface damage in the constituent phases, particularly at the composite–metal interface, to assess the structural integrity. The temperature contours obtained from the proposed oblique model are summarized in Figure 10.

In the GFRP phase, the affected depth appears to be minimally sensitive to the cutting speed but shows a strong dependence on the sequencing of the phases. In the case of cutting the GFRP → Al stack, the affected depth in the GFRP phase is 2.34±0.075 times greater than that observed during cutting of the Al → GFRP stack. When cutting starts with the Al phase, the metallic alloy induces relatively more severe mechanical damage at the beginning of the process than in the case of Al → GFRP cutting, due to plastic flow in the secondary shear zone. Locally, at the interface, chip formation then leads to premature removal of the hottest elements that were critically damaged mechanically. Consequently, in the case of Al → GFRP cutting, the prematurely removed hottest elements will not ensure continuous and deep heat transfer into the GFRP phase. This explains the shallow thermally affected depth in the case of Al → GFRP cutting, as reflected by the temperature contours observed in the composite phase. However, when comparing the affected depth obtained from the two cutting configurations at the interface, the resulting ratio is much lower than that observed for the GFRP plate.

The thermal damage induced when cutting the hybrid starting with the Al phase (Figure 10b) is 1.15±0.04 times higher than that recorded when starting with the GFRP phase (Figure 10a). This difference in the thermal process, which is closely correlated with mechanical damage, confirms (i) firstly, the importance of the oblique cutting model compared to the traditional orthogonal cutting model, which cannot detect this flow orientation, and (ii) secondly, the necessity of considering the inclination angle $\lambda_{s}$ in the oblique cutting model, a factor that has not been addressed in the literature. This last point represents the major innovation of this study.

## 5. Conclusions

This study presents a novel thermomechanical approach to examine the integrity of GFRP/Al hybrid structures subjected to single-shot drilling. It aims to understanding thermal damage mechanisms within the hybrid composites in order to refine the control over the material removal process at the interface. The proposed model was described by a user-defined subroutine VUMAT interacting with a VDFLUX. The dynamic chip formation resulting in drilling of composite stack was simulated referring to double-inclination oblique cutting configuration. Typically, the effects of the cutting speed and stacking arrangement on sub-surface damage and temperature evolution at the GFRP/Al interface were discussed. The key findings are summarized as follows:

- Increasing spindle speed significantly affects the temperature distribution and thermal subsurface damage within the composite structure. When the tool engages first the GFRP phase (GFRP → Al), temperature stabilizes at a speed of approximately 308 m/min. However, when the tool engages first the Al phase (Al → GFRP), stabilization is achieved at about 471 m/min.
- The choice of cutting arrangement critically affects the temperature distribution and subsurface damage. It was revealed that attacking from the GFRP phase results in higher interface temperatures than when attacking from the aluminum side. This is likely due to GFRP’s lower thermal conductivity compared to that of aluminum, which restricts heat dissipation and causes higher temperature buildup in the GFRP layer during cutting.
- If compared with drilling tests, the proposed model shows high reliability in predicting drilling behavior regardless of the stacking arrangement. The findings make it possible to derive an exponential temperature law, which not only predicts temperature changes versus the cutting speed, but also identifies thermal saturation at the interface. This make it possible to reveal the critical cutting speed range to avoid when drilling such a structure.
- The heat transfer between constitutive phases influences the subsurface damage at the interface. Heat localization is typically detected at the interface due to temperature overlaps exceeding the glass transition point $\left(T>T_{g}\right)$. Heat generated in the metallic phase flows to the GFRP phase and elevates the temperature throughout the interface. The temperature gap between the phases yields a severe discontinuity along the interface, which promotes failure initiation and ultimately compromises the integrity of the hybrid structure during drilling.

## Figures and Tables

**Table 1:** Hashin damage criteria for modeling failure in the GFRP Phase [40].

Parameters	Symbol	Value
Density	$\rho \;\left({\mathrm{Kg}\;m}^{-3}\right)$	1230
Longitudinal Young’s modulus in-plan	$E_{11}\left(\mathrm{GPa}\right)$	48
Transverse Young’s modulus in-plan	$E_{22}\left(\mathrm{GPa}\right)$	12
Transverse Young’s modulus normal-to-plan	$E_{33}=E_{22}\;\left(\mathrm{GPa}\right)$	12
In-plane shear modulus	$G_{12}\;\left(\mathrm{GPa}\right)$	6
Shear modulus in plane 1–3	$G_{13}=G_{12}\;\left(\mathrm{GPa}\right)$	6
Shear modulus in plane 2–3	$G_{23}\;\left(\mathrm{GPa}\right)$	4
In-plane Poisson’s ratio	$\nu_{12}$	0.28
Transverse Poisson’s ratio in 1–3 plan	$\nu_{13}=\nu_{12}$	0.28
Transverse Poisson’s ratio in 2–3 plan	$\nu_{23}$	0.35
Longitudinal tensile strength in-plan	$X_{t}\;\left(\mathrm{MPa}\right)$	1200
Longitudinal compression strength in-plan	$X_{c}\;\left(\mathrm{MPa}\right)$	800
Transverse tensile strength in-plan	$Y_{t}\;\left(\mathrm{MPa}\right)$	59
Transverse tensile strength normal-to-plan	$Z_{t}=Y_{t}\;\left(\mathrm{MPa}\right)$	128
Transverse compression strength in-plan	$Y_{c}\;\left(\mathrm{MPa}\right)$	59
Transverse compression strength normal-to-plan	$Z_{c}=Y_{c}\;\left(\mathrm{MPa}\right)$	128
In-plane shear strength	$S_{12}\;\left(\mathrm{MPa}\right)$	25
Transverse shear strength in plane 1–3	$S_{13}=S_{12}\;\left(\mathrm{MPa}\right)$	25
Transverse shear strength in plane 2–3	$S_{23}\;\left(\mathrm{MPa}\right)$	25
In-plane longitudinal thermal conductivity	$\lambda_{11}\;\left({\mathrm{Wm}}^{-1}K^{-1}\right)$	2.38
Transverse thermal conductivity in-plane	$\lambda_{22}\;\left({\mathrm{Wm}}^{-1}K^{-1}\right)$	0.39
Transverse thermal conductivity normal-to-plane	$\lambda_{33}\;\left({\mathrm{Wm}}^{-1}K^{-1}\right)$	0.39
Specific heat	$C_{p}\;\left({\mathrm{JKg}}^{-1}K^{-1}\right)$	700

**Table 2:** Mechanical properties and Johnson–Cook parameters used for Al alloy [41].

Parameters	Symbol	Value
Density	$\rho \;\left({\mathrm{Kg}\;m}^{-3}\right)$	2770
Young’s modulus	$E\;\left(\mathrm{GPa}\right)$	73
Poisson ratio	ν	0.33
Melting temperature	$T_{m}\;\left(℃\right)$	520
Room temperature	$T_{0}\;\left(℃\right)$	25
Thermal conductivity	$\lambda_{11}\;\left({\mathrm{Wm}}^{-1}K^{-1}\right)$	120
Thermal expansion coefficient	$\alpha \;\left({℃}^{-1}\right)$	$24.7\times 10^{-6}$
Specific heat	$C_{p}\;\left({\mathrm{JKg}}^{-1}K^{-1}\right)$	875
Viscoplastic parameters	$A\;\left(\mathrm{MPa}\right)$	352
	$B\;\left(\mathrm{MPa}\right)$	440
	$C\;\left(\mathrm{MPa}\right)$	0.0083
	n	0.42
	m	1
Damage parameters	$D_{1}$	0.13
	$D_{2}$	0.13
	$D_{3}$	1.5
	$D_{4}$	0.011
	$D_{5}$	0

**Table 3:** Cutting parameters considered for the simulation of GFRP/Al cutting.

Parameters	Symbol	Value
Tool rake angle	$\gamma \;\left({}^{\circ}\right)$	11
Tool clearance angle	$\alpha \;\left({}^{\circ}\right)$	5
Inclination angle	$\lambda_{s}\;\left({}^{\circ}\right)$	12
Chip flow angle	${µ}_{c}\;\left({}^{\circ}\right)$	11
Tool-tip radius	$r_{c}\;\left(\mu m\right)$	5
Specimen size	$\left({\mathrm{mm}}^{3}\right)$	3×3×0.4
Cutting speed	$v_{c}\;\left({m\;\min}^{-1}\right)$	71–94–119–142
Depth of cut	$a_{p}\;\left(\mu m\right)$	6
Fiber orientation	$\theta \;\left({}^{\circ}\right)$	0
Friction coefficient	μ	0.3

**Table 4:** Temperature distribution vs. cutting speed: cutting GFRP → Al.

Cutting Speed	GFRP Plate	Al Plate
$v_{c}=71\;m\cdot \min^{-1}$	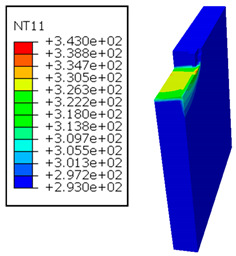	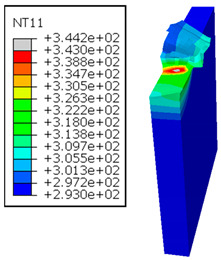
$v_{c}=94\;m\cdot \min^{-1}$	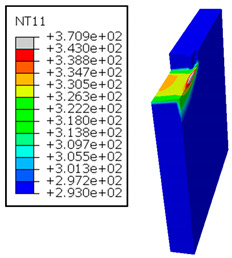	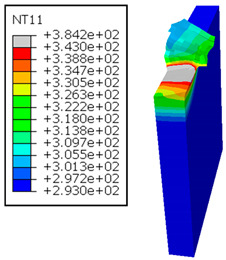
$v_{c}=119\;m\cdot \min^{-1}$	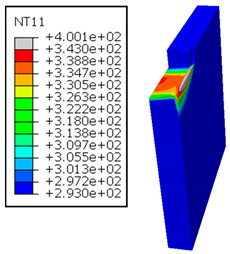	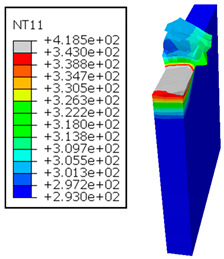
$v_{c}=142\;m\cdot \min^{-1}$	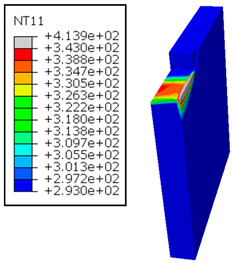	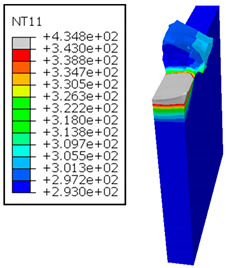

**Table 5:** Temperature distribution vs. cutting speed: cutting Al → GFRP.

	Al Plate	GFRP Plate
$v_{c}=71\;m\cdot \min^{-1}$	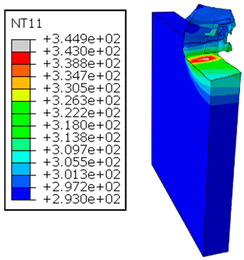	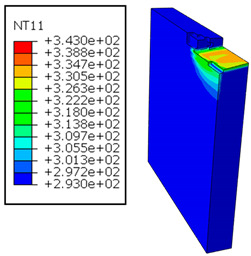
$v_{c}=94\;m\cdot \min^{-1}$	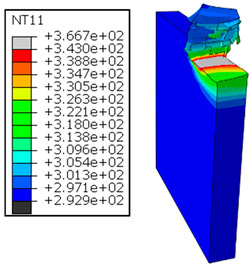	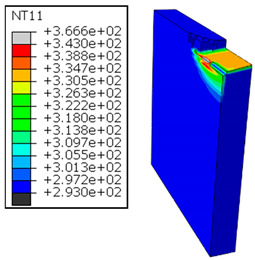
$v_{c}=119\;m\cdot \min^{-1}$	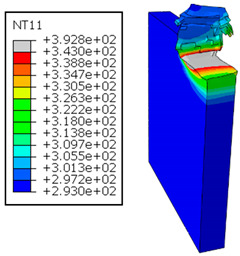	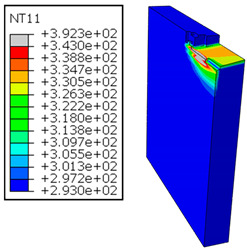
$v_{c}=142\;m\cdot \min^{-1}$	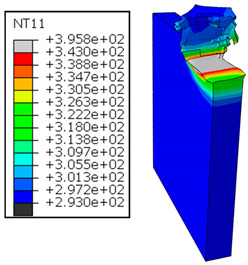	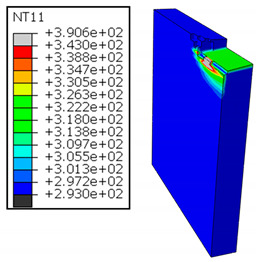

## Data Availability

The original contributions presented in the study are included in the article. Further inquiries can be directed to the corresponding author.
